# Assessment of time intervals in the pathway to oral cancer diagnosis in north-westerm Spain. Relative contribution of patient interval

**DOI:** 10.4317/medoral.21676

**Published:** 2017-06-04

**Authors:** Pablo Varela-Centelles, José-Luis López-Cedrún, Jacinto Fernández-Santromán, Pablo Álvarez-Nóvoa, Ramón Luaces-Rey, María-Josefa Pombo-Castro, María-Pía López-Jornet, Juan Seoane

**Affiliations:** 1 Primary Care Clinics. CS Praza do Ferrol. EOXI Cervo, Lugo, e Monforte de Lemos. Galician Health Service. Lugo. Spain; 2 Dept of Medical-Surgical Specialities. University of Santiago de Compostela. Santiago de Compostela (A Coruña). Spain; 3 Service of Oral and Maxillofacial Surgery. Complexo Hospitalario Universitario de A Coruña (CHUAC). Galician Health Service. A Coruña. Spain; 4 Service of Oral and Maxillofacial Surgery. POVISA Hospital. Vigo. (Pontevedra). Spain; 5 Faculty of Medicine. University of Murcia. Murcia. Spain

## Abstract

**Background:**

Despite continuous advances in diagnosis and therapy, oral cancers are mostly diagnosed at advanced stages with minor survival improvements in the last two decades. Both phenomena have been attributed to delays in the diagnosis. This study aims at quantifying the time elapsed until definitive diagnosis in these patients and the patient interval’s contribution.

**Material and Methods:**

A hospital-based, ambispective, observational study was undertaken on incident cases with a pathological diagnosis of oral squamous cell carcinoma recruited during 2015 at the Oral and Maxillofacial Surgery services of CHUAC (A Coruña) and POVISA (Vigo) hospitals.

**Results:**

74 consecutive oral cancer patients (59.5% males; median age: 65.0 years (IQ:57-74)) were studied. Most cases (52.7%; n=39) were at advanced stages (TNM III-IV) at diagnosis. The period since first sign/symptom until the patient seeks health care was the longest interval in the pathway to diagnosis and treatment (median: 31.5 days; IQR= 7.0 – 61.0) and represents >60% of the interval since symptom onset until referral to specialised care (pre-referral interval). The average interval assigned to the patient resulted to be relatively larger than the time elapsed since the patient is seen at primary care until a definitive diagnosis is reached (diagnostic interval). Median of the referral interval for primary care professionals: 6.5 days (IQR= 0.0 – 49.2) and accounts for 35% (19% - 51%) of the diagnostic interval.

**Conclusions:**

The patient interval is the main component of the pathway to treatment since the detection of a bodily change until the definitive diagnosis. Therefore, strategies focused on risk groups to shorten this interval should be implemented in order to ease an early diagnosis of symptomatic oral cancer.

** Key words:**Oral cancer, early diagnosis, diagnostic delay, time interval, time to diagnosis, Aarhus statement.

## Introduction

Despite the continuous advances in the fields of diagnosis and therapy, oral cancers are still diagnosed mostly at advanced stages (III-IV) and the improvements in terms of patient survival have been very minor (5%) in the last two decades (1,2). Both phenomena have been put down to delays in the diagnosis of the disease (3,4). This hypothesis has been confirmed by different meta-analitical studies which unveiled the relationship between diagnostic delay and advanced disease (5) and a moderate impact on survival to head and neck carcinomas (6).

For over 75 years the culpability for this delay has been attributed either to the patient or both to patients and physicians (7). In the particular case of oral cancer, some methodologically heterogeneous studies using a range of different arbitrary criteria to disclose delayed cases have identified the patient, the clinician, and the health system to be responsible for the delays in diagnosis (8,9).

This overall diagnostic delay (period elapsed between the first symptom or sign and the definitive histological diagnosis) has been extensively studied by several research groups that have independently identified various time lapses with a potential role in delayed diagnoses, namely patient delay, scheduling delay or primary care delay, appointment delay (specliased care diagnostic interval), and medical specialist delay (10-12). All these reports based upon the idea of apportioning blame for diagnostic delay, -putting aside their hypothetical legal consequences- have proved unable both to monitor the process until the final diagnosis of symptomatic oral cancer and to render consistent and reliable results (8).

In an attempt to ease comparability among studies and to standardise the key points (time intervals since detection of a bodily change until treatment is started), the use of the research model “model of pathways to treatment” has been encouraged together with a specific methodology aimed at minimising potential biases (13). Recently, a systematic review has identified the actual periods where a delay may be found in the particular path of a patient with symptomatic oral cancer (14). Also, the use of term “diagnostic delay” is not recommended for research on this topic (13,14).

Despite information on the aforementioned topics would be paramount to identify targets for intervention when pursuing an early oral cancer diagnosis, there are no reports quantifying the relative contribution of these time intervals to the time to diagnosis designed within the consensus theoretical framework (The Aarhus Statement Model) (13).

Therefore, the aim of this investigation was to quantify the time intervals elapsed until definitive diagnosis in patients suffering from symptomatic oral cancer, as well as to assess the relative length of the patient interval in terms of overall diagnostic delay.

## Material and Methods

A hospital-based, ambispective, observational study was undertaken where the prospective component starts at the moment of the patient’s first contact with the specialist who will be treating his/her disease. The subjects of the study were incident cases with a pathologically confirmed diagnosis of oral squamous cell carcinoma (OSCC) recruited during 2015 at the Oral and Maxillofacial Surgery services of CHUAC (A Coruña) and POVISA (Vigo) hospitals. These hospitals serve a total of 356 primary care centres in Galicia (North-western Spain). The exclusion criteria were: prevalent/recurrent cases, second primary tumours, multiple carcinomas, or patients treated at hospitals outside of the public health service network.

The model of pathways to treatment was chosen to establish the time intervals since the detection of a bodily change to the definitive (pathological) diagnosis of oral squamous cell carcinoma (13-15). The patient-related interval, together with the primary care and diagnostic intervals were also considered, as well as their relative contribution to the overall time to definitive diagnosis. In order to minimise memory bias, particularly those related to the date of first symptoms detection and date of first presentation, patient self-reported information was checked against patient’s relatives and clinical records both at primary care and hospital levels. Relevant dates were obtained from the patient by means of a structured, face-to-face conversation with the surgeon as part of the routine clinical interview for hospital records.

**Figure 1:**
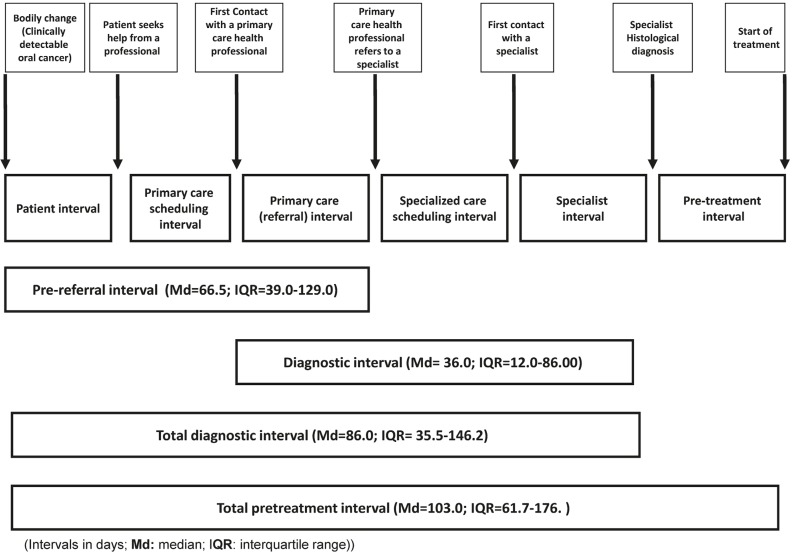
Adaptation of the pathways to treatment model to the particularities of oral cancer.

Definition of intervals: In compliance with the Aarhus Statement (13,14), we considered the time intervals outlined by the fol-lowing events: bodily change (sign/symptom onset), first seen at primary care level, referral for specialised care, and definitive diagnosis. Additional time lapses identified as potential sources of patient delay (10,11,13), and appointment delay (14,16) were also studied. A pre-referral interval (16) (patient interval + primary care interval), a diagnostic interval (16) (time from first seen in primary care to definitive diagnosis), and a total diagnostic interval (14,16,17) were also calculated (Fig. 1). Participants gave their informed consent before entering the study, which was approved by the relevant committee of ethics in research (No. 2014/604).

- Statistical analysis

Statistical analysis was undertaken using the SPSS+ 15.0 statistical package (Chicago, IL, USA). The mean and the median (Md) were used as central trend statistics, and the interquartile range and the 90th centile as spread indicators, when describing the time intervals (days). The ratio between means and medians of patient interval to primary care interval, to pre-referral interval, to diagnostic interval, and to total diagnostic interval were also calculated, assuming the conditions for using the test.

## Results

A convenience sample of 74 OSCC patients (59.5% males; median age: 65.0 years (IQ:57-74)) was studied. The most frequently affected sites were the tongue (n=38; 51.4%) and the palate (n=10; 13.5%), followed by the floor of the mouth and gingiva (n=4; 5.4%). Other locations, altogether, account for 23% of the sample (n=17). Most cases (52.7%; n=39) were at advanced stages (TNM III-IV) at the time of diagnosis.

The period since the first cancer-related sign/symptom is detected until the patient demands an appointment at primary care resulted to be the longest interval in the subject’s pathway to diagnosis and treatment (median: 31.5 days; IQR= 7.0 – 61.0) and accounts for more than 60% of the interval since the symptoms onset until the patient is referred for specialised care (pre-referral interval). Besides, the average interval assigned to the patient resulted to be relatively larger than the time elapsed since the patient is seen at primary care until a definitive diagnosis is reached (diagnostic interval). The referral interval assigned to primary care professionals (dentists and/or physicians) elicited a median of 6.5 days (IQR= 0.0 – 49.2) and accounts for 35% (19% - 51%) of the average diagnostic interval. The other intervals in the pathway to diagnosis analysed in this study have been significantly shorter: the specialist interval (oral and maxillofacial surgeon) showed a median of 6.0 days (IQR= 4.0 – 11.25); the intervals of appointment and scheduling at primary care (median: 1.5 days; IQR= 0.00 – 8.5) and at a specialised level (median: 1.5 days; IQR= 6.0 – 17.5) were not relevant in terms of the overall time to diagnosis ( Table 1, Table 2).

**Table 1:**
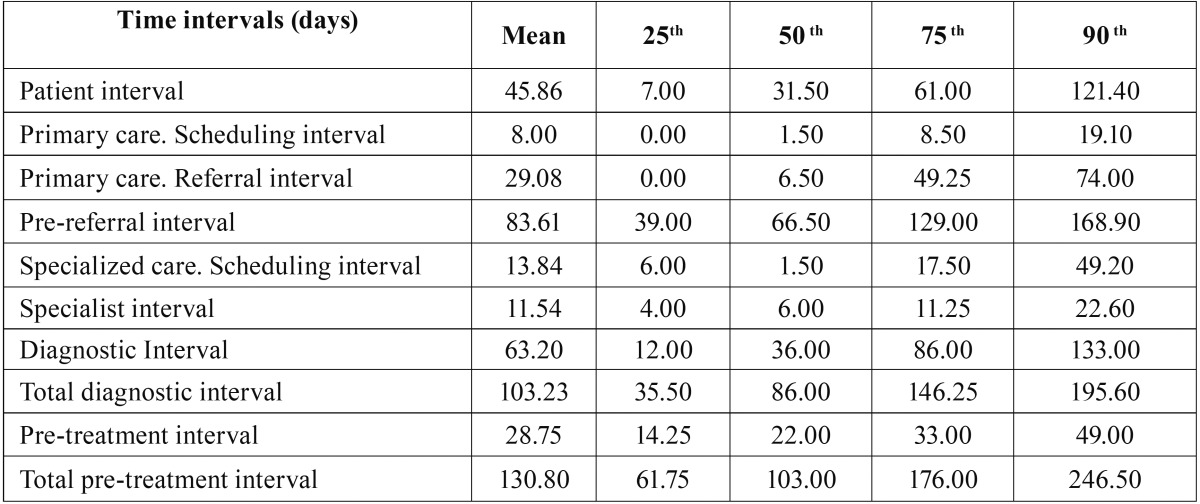
Time intervals (days) in the sample until treatment is started.

**Table 2:**
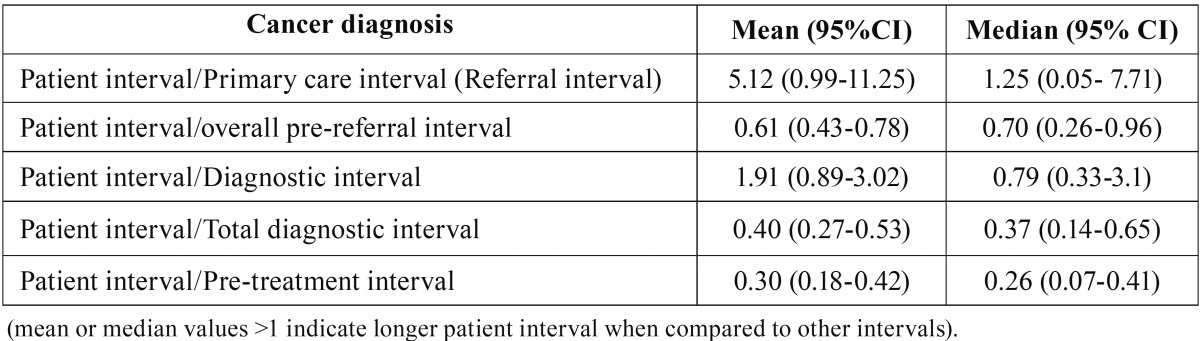
Mean – median ratio of the patient interval over other time intervals.

## Discussion

This study is the first investigation reporting quantitative data on each of the time intervals symptomatic OSCC patients go through to reach a definitive diagnosis, according to the “model of pathways to treatment” (13). Although this model would permit prioritisation of interventions for early diagnosis, there are some limitations in our research that have to be taken into account, mostly linked to the sample itself and to the fact it is a hospital-based study. On the other hand, a selection bias is highly unlikely due to the absence of drop-outs, and also because the features of the sample are not different from the general characteristics of cancer patients at diagnosis (1). The main strengths of our study rely on the use of the guidelines for improving design and reporting studies in early cancer diagnosis (Aarhus statement) (13), with clearly defined start- and endpoints within the theoretical framework in a prospective design, which increases the quality of the data collected. Moreover, and to prevent a potential recall bias, the information provided by the patients were double-checked against patient’s relatives and clinical records at both primary and specialised care levels.

In our series, total diagnostic delay has shown values close to the reported from Japan (24), and shorter time intervals than those reported from Iran (20). In this sense, the total pre-treatment interval found in out study resulted to be markedly lower than reported by a narrative review summarizing data from patients in Germany, UK, and USA, where values ranging from 5 to 6 months or longer were recorded (8).

Assuming that large intervals to treatment result in poor outcomes it is paramount to know the relative contribution of each individual interval to the whole process of reaching treatment (total pre-treatment interval) in order to target potential interventions (13,14).

In this sense, the analysis of the patient-related delay (classically defined as “patient diagnostic delay”) has produced equivocal results (8,9): different papers published in the last decade report a median <1.5 months (18,19), whereas other groups described much longer intervals (20), ranging from 3.5 to 4 months where the patient delay prevails over the professional and health-system delays (8). This circumstance may well be explained by the lack of specificity of the signs and symptoms of OSCC, the difficulty in identifying certain symptoms considered as potentially dangerous (12), and also by the differences in socio-cultural environments which condition symptom interpretation and become determinants linked to longer patient intervals (22).

Our results show a median of the patient interval of 31.5 days, very close to the 30 days reported by the National Audit of Cancer Diagnosis in Primary Care (UK) and also to other reported series from Finland, which share similar socio-economic contexts despite the evident geographical disparities (16,18,19). The patient interval depends greatly on tumour site, and patients with oropharyngeal, laryngeal, oesophageal, and neck carcinomas show the longest patient intervals (16).

Bearing in mind that a longer pre-referral interval (patient + primary care interval) is a risk factor for advanced disease and mortality from oral cancer (6) it is fundamental to know the relative length of both intervals. Our study has shown a clear predominance of the patient interval over that of the primary care professional, as has been previously described for other sites (melanoma, breast, testicular, vulval, cervical, and laryngeal carcinomas) (16).

The primary care interval is quite stable for each location, and it is related to the number of consultations at the primary care level. This interval tends to be shorter for those neoplasms showing visible or palpable lesions, such as breast, endometrium, and vulva (16). The primary care interval in our series (Md=6.5 days) is very similar to those reported by other oral cancer series (11,24) and close to the ones reported for thyroid and laryngeal cancers (16). However, despite oral cancer is considered a carcinoma “easy-to suspect” after presentation in primary care, where early signs such as changes in colour and texture or event the presence of precursor lesions (DOPM) (21) are clear warning clues, some series have reported large primary care intervals with means and medians about one month (25) or even longer (1.9 months) in India (26).

Several contributing factors, like system factors (health care policy) and patient factors often act together in different time intervals, as occurs at the primary care and the specialised care scheduling intervals, with a median of just 1.5 days in our series. This is relevant, as the primary care scheduling interval has been classically computed as “patient delay” although it is also conditioned by the availability and accessibility of the healthcare system (8).

-Practical implications for research and health policy

In order to avoid terminological inconsistencies and to ease comparisons between studies on early cancer diagnosis, it is strongly recommended to follow the criteria of the Aarhus Statement guidelines. The adaptation of the “pathways to treatment” depicted in figure 1 may be useful for this purpose.

The patient interval is the main component of the total diagnostic interval, and it is proportionally larger than the interval assigned to Primary Care, and even longer than the diagnostic interval (from the first consultation at primary care level to definitive diagnosis). Paradoxically, a great number of interventions aimed at increasing oral cancer knowledge among general dentists and physicians have been undertaken to shorten the patient’s referral period to specialised care, but oral cancer patient-centered interventions are very scarce (27,28). Actually, patients are the principal target for interventions, which should be focused at increasing public awareness of the disease. In this sense, and despite the reported transitory effects, mass media information campaigns (radio and television, newspapers, Internet, and even advertisements on billboards) offer opportunities for saving lives and may be useful for raising cancer awareness. However, only information leaflets have proved a significant effect on raising the long-term oral cancer knowledge and demonstrated a subsequent impact on disease awareness among the public (28). Besides, it should be taken into account that oral cancer awareness is particularly deficient among high-risk groups and those with lower socio-economic status, and also that community-based educational interventions have demonstrated poor effectivity (28), perhaps due to a poor adaptation to the socio-cultural context of at-risk population.

Our results also show a margin for improvement in the diagnostic interval, where some advantages can be taken from previous experiences. Thus, interventions aimed at diminishing the primary care interval (such as the NICE guidelines) have proved effective in reducing the diagnostic interval for head and neck carcinomas (29). In the same way, an adequate prioritisation of patients with head and neck carcinomas has been able to significantly shorten the interval from referral to treatment (30).

## Conclusions

The patient interval is the main component of the pathway to treatment since the detection of a bodily change until the definitive diagnosis. Therefore, strategies focused on risk groups to shorten this interval should be implemented in order to ease an early diagnosis of symptomatic oral cancer.
